# Non‐adjacent Dependency Learning in Humans and Other Animals

**DOI:** 10.1111/tops.12381

**Published:** 2018-09-08

**Authors:** Benjamin Wilson, Michelle Spierings, Andrea Ravignani, Jutta L. Mueller, Toben H. Mintz, Frank Wijnen, Anne van der Kant, Kenny Smith, Arnaud Rey

**Affiliations:** ^1^ Institute of Neuroscience Newcastle University; ^2^ Department of Cognitive Biology University of Vienna; ^3^ Research Department Sealcentre Pieterburen; ^4^ Artificial Intelligence Lab Vrije Universiteit Brussel; ^5^ Institute of Cognitive Science University of Osnabrueck; ^6^ Departments of Psychology and Linguistics University of Southern California; ^7^ Utrecht Institute of Linguistics OTS Utrecht University; ^8^ Department of Linguistics University of Potsdam; ^9^ Centre for Language Evolution University of Edinburgh; ^10^ CNRS Aix‐Marseille University

**Keywords:** Non‐adjacent dependency, Artificial grammar, Structured sequence processing, Human, Infant, Nonhuman animal, Primate

## Abstract

Learning and processing natural language requires the ability to track syntactic relationships between words and phrases in a sentence, which are often separated by intervening material. These nonadjacent dependencies can be studied using artificial grammar learning paradigms and structured sequence processing tasks. These approaches have been used to demonstrate that human adults, infants and some nonhuman animals are able to detect and learn dependencies between nonadjacent elements within a sequence. However, learning nonadjacent dependencies appears to be more cognitively demanding than detecting dependencies between adjacent elements, and only occurs in certain circumstances. In this review, we discuss different types of nonadjacent dependencies in language and in artificial grammar learning experiments, and how these differences might impact learning. We summarize different types of perceptual cues that facilitate learning, by highlighting the relationship between dependent elements bringing them closer together either physically, attentionally, or perceptually. Finally, we review artificial grammar learning experiments in human adults, infants, and nonhuman animals, and discuss how similarities and differences observed across these groups can provide insights into how language is learned across development and how these language‐related abilities might have evolved.

## Abstract 2

Wilson et al. focus on one class of AGL tasks: the cognitively demanding task of detecting non‐adjacent dependencies (NADs) among items. They provide a typology of the different types of NADs in natural languages and in AGL tasks. A range of cues affect NAD learning, ranging from the variability and number of intervening elements to the presence of shared prosodic cues between the dependent items. These cues, important for humans to discover non‐adjacent dependencies, are also found to facilitate NAD learning in several nonhuman animal species.

## 1 Introduction

A central feature of syntactic processing is the ability to track structural relationships between words and phrases in a sentence. However, language has a hierarchical structure; hence, syntactic relationships exist not only between *adjacent words* but also across longer distances, requiring the joint processing of words separated by intervening material. Tracking such *nonadjacent dependencies* is more cognitively complex than processing the relationships between adjacent words, minimally placing additional demands on working memory. Moreover, the ability to detect nonadjacent dependencies is a crucial prerequisite for learning and processing some more complex syntactic relationships (like center embedded structures). Understanding how we learn to detect and process nonadjacent dependencies, and the ontogenetic and evolutionary origins of these abilities, represents an important challenge in understanding key aspects of language processing, acquisition, and evolution.

A nonadjacent dependency is a relationship between two temporally or spatially separated elements, which cannot be explained simply by the occurrence of multiple pairs of adjacent relationships. For example, in English, nonadjacent dependencies can be observed in tense agreement (e.g., “*Is* [talk]*ing*,” “*Has* [talk]*ed*”) or subject‐verb agreement (e.g., “*the* *dog* *[down the street]* *barks*” vs. “*the* *dogs* *[down the street]* *bark*”). In both of these cases the dependent elements can be separated by intervening material, and one must track the nonadjacent dependency, holding the first element in memory until the appropriate point later in the sequence.

One productive way to study how dependencies are learned and processed is to use artificial grammar learning paradigms and structured sequence processing tasks. These experiments typically test participants' abilities to learn relationships between specific elements in sequences of auditory or visual stimuli (Reber, 19671967). Such tasks do not rely on existing semantic or syntactic knowledge and are therefore an important tool with which to isolate and study the cognitive and neurobiological systems that support how specific aspects of syntax may be learned and processed (Petersson, Folia, & Hagoort, 20122012). Furthermore, these tasks seem to tap into language‐relevant capacities, as evidenced by correlations between nonadjacent dependency learning and natural language processing in adult humans (Misyak, Christiansen, & Tomblin, 20102010), and by the fact that nonadjacent dependency learning is impaired in individuals with specific language impairment (Hsu, Tomblin, & Christiansen, 20142014) or familial risk of dyslexia (Kerkhoff, De Bree, De Klerk, & Wijnen, 20132013). Finally, as these tasks do not require language, they can be used to test preverbal infants, to inform us about language acquisition (e.g., Gómez, 20022002; Saffran, Aslin, & Newport, 19961996), and nonhuman animals, which can provide insights into language origins and evolution (e.g., Fitch & Hauser, 20042004; Newport, Hauser, Spaepen, & Aslin, 20042004).

In a seminal study of nonadjacent dependency learning, Gómez (20022002) presented adult participants and 18‐month‐old infants with sequences of nonsense words (e.g., “*pel wadim rud*,” “*vot kicey jic*”) containing a nonadjacent dependency in which the first word predicted the final word (i.e., “*pel*” predicts “*rud*”; “*vot*” predicts “*jic*” regardless of the identity of the middle word in the sequence). Participants were then tested with sequences that were either consistent or inconsistent with this dependency (e.g., “*pel wadim rud*” vs. “*pel wadim* *jic*”). Gómez found that in some conditions (see below) adults and infants are able to learn these nonadjacent dependencies, discriminating between sequences which conform to or violate the dependency. This result has been confirmed in further experiments involving adults (e.g., Frost & Monaghan, 20162016; Gómez, 20022002; Pena, Bonatti, Nespor, & Mehler, 20022002; van den Bos, Christiansen, & Misyak, 20122012; Vuong, Meyer, & Christiansen, 20162016) and infants (e.g., Gómez, 20022002; Gómez & Maye, 20052005) as well as nonhuman animals (e.g., Milne et al., 20162016; Newport et al., 20042004; Ravignani, Sonnweber, Stobbe, & Fitch, 20132013; Sonnweber, Ravignani, & Fitch, 20152015; Versace, Rogge, Shelton‐May, & Ravignani, 20172017), although successful learning only occurs under certain conditions (see Section** 33, below).

In this article, we first characterize different types of nonadjacent dependencies, and how they relate to nonadjacent syntactic relationships in natural languages. We summarizse the evidence for nonadjacent dependency learning in human adults and infants, and nonhuman animals, review the conditions under which learning does and does not occur, and discuss the insights these studies provide into the development and evolution of these abilities.

## 2 Characterizing different types of nonadjacent dependency

The simplest form of structural dependency that might appear within a sentence is a syntactic relationship between two adjacent words. In artificial grammar learning tasks, this type of adjacent dependency might be present between two sequentially presented elements “A” and “B” (see Fig. 11i). These adjacent dependencies are present in most artificial grammar learning studies and are rapidly learned by human adults and infants, and nonhuman animals (e.g., Chen & ten Cate, 20152015; Fitch & Hauser, 20042004; Gebhart, Newport, & Aslin, 20092009; Pacton, Sobaco, & Perruchet, 20152015; Reber, 19671967; Saffran et al., 19961996; Wilson, Smith, & Petkov, 20152015). By contrast, a nonadjacent dependency represents a relationship between items that are separated by one or more intervening elements in a sequence (Fig. 11ii and iii, e.g., Gómez, 20022002; Newport et al., 20042004). Both adjacent and nonadjacent dependencies might require participants to learn the relationship between the same two elements (“A” and “B” in Fig. 11), yet the cognitive and memory demands might vary in relation to the distance over which this dependency must be processed. Such nonadjacent relationships might appear within a longer sequence (Fig. 11iv), removing the opportunity to rely on the edges of the sequence to identify key dependencies (e.g., Endress, Carden, Versace, & Hauser, 20102010; Endress, Nespor, & Mehler, 20092009; Grama, Wijnen, & Kerkhoff, 20132013). Finally, many artificial grammar learning paradigms are not designed specifically to assess the learning of nonadjacent dependencies, but nevertheless require this ability as a prerequisite to process more complex dependencies. For example, many studies have used a grammar of the form A^*n*^B^*n*^ to assess different forms of hierarchical sequence processing (e.g., center‐embedding, crossed dependencies, which are present in some natural languages [Bach, Brown, & Marslen‐Wilson, 1986], Fig. 11v,vi), which require the participant to process multiple adjacent and nonadjacent dependencies between “A” and “B” elements (De Vries, Christiansen, & Petersson, 20112011; De Vries, Petersson, Geukes, Zwitserlood, & Christiansen, 20122012). While these more complex tasks bring additional cognitive demands, all of these tasks require the ability to identify and process nonadjacent dependencies, which represents an important prerequisite to aspects of syntax processing.

**Figure 1:**
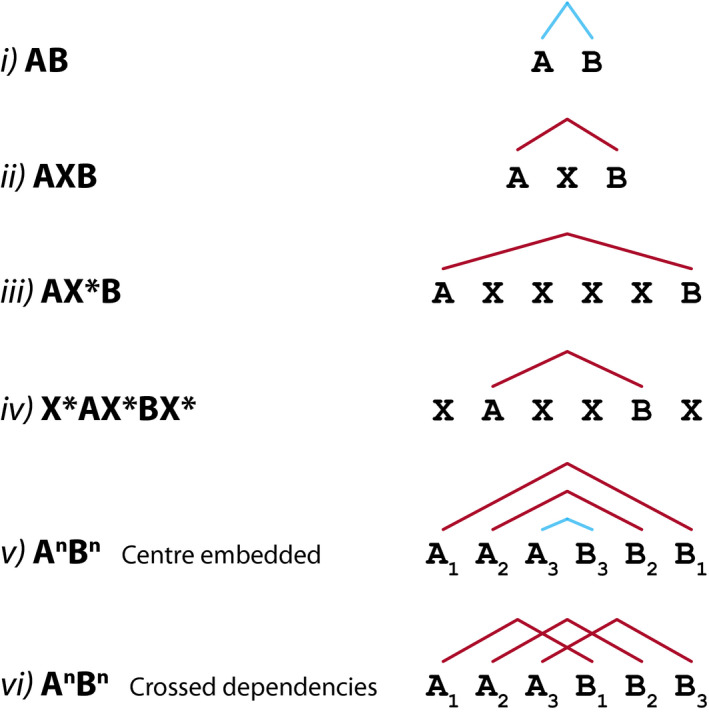
Adjacent and nonadjacent dependencies in several commonly used artificial grammar structures. Adjacent dependencies are shown in blue and nonadjacent dependencies are shown in red. More complex grammars (e.g., A^*n*^B^*n*^) go beyond the requirement to learn a single nonadjacent dependency at a time and require several dependencies to be processed simultaneously.

Sequence learning experiments have also manipulated the nature of the relationships between the dependent elements. A simple type of dependency is the relationship between two identical elements, and it is known as an identity relation (see Fig. 22i). This type of identity relation is sometimes observed in natural language. For example, agreement systems in Bantu languages often involve the appearance of an identical prefix on words which agree, as in Swahili, for example, the “*ki*” prefix in “*Ki‐kapu ki‐dogo ki‐me-fika*” (“the little basket arrived”). It has been suggested that this transparent means of signaling agreement in these languages allows them to sustain unusually complex agreement systems arising from a large number of noun classes (Demuth & Weschler, 20122012). In an artificial grammar learning task, a nonadjacent dependency of this type might take the form “AXA,” where a dependency exists between two identical sequence elements. Learning and processing this dependency requires only that the initial element be held in memory, and that incoming stimuli be compared to this memory representation.

**Figure 2:**
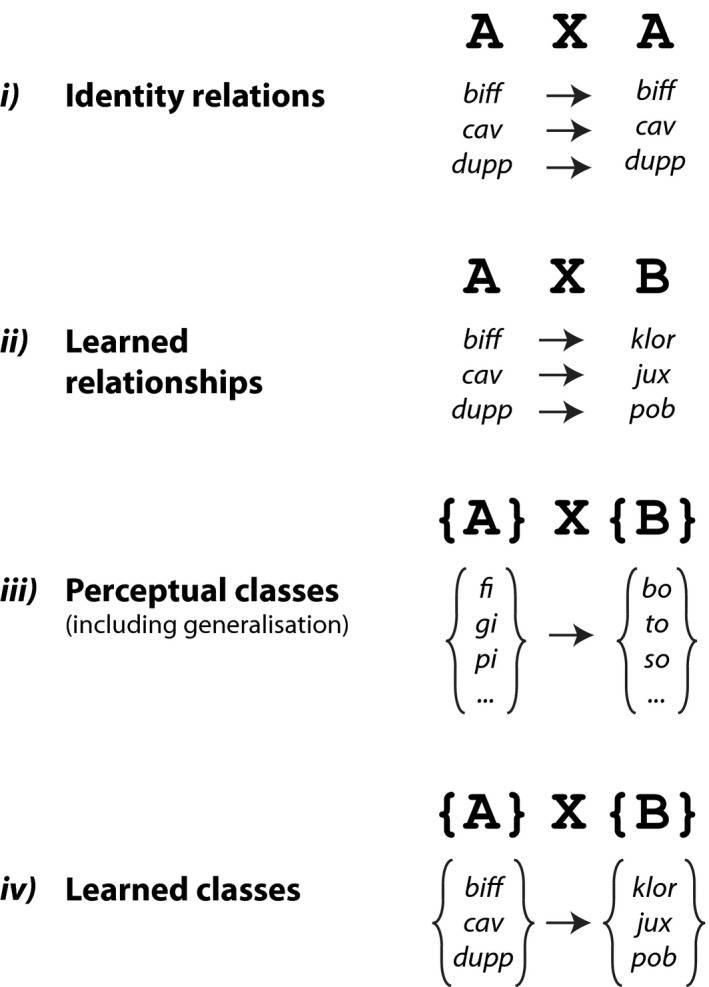
Different types of stimulus classes used in nonadjacent dependency learning tasks. Several varieties of nonadjacent dependencies can be assessed in artificial grammar learning studies. These include (i) identity relations between specific elements (as can be seen in certain Bantu languages); (ii) learned relationships between specific elements (as in English tense agreement); (iii) relationships between perceptual classes; (iv) relationships between learned classes (similar to dependencies between syntactic word categories; for example, nouns and verbs). See Endress and Bonatti (20072007).

Bantu languages like Swahili notwithstanding, most languages rarely require us to detect repetitions of the same element within a sentence (as in Fig. 22i), but rather to track dependencies between different words or part‐words that share little phonological resemblance, based on learned relationships. As in the previous example of tense agreement, one must learn the relationship between “*is*” and the suffix “*_ing*” based on their co‐occurrence, before one is able to process this dependency or detect ungrammatical sentences (e.g., Friederici, Mueller, & Oberecker, 20112011). This has been investigated using artificial grammars of the form “AXB” (Fig. 22ii). In such studies the first element predicts a different final element, and the dependent elements are related only by an arbitrary pairwise association, which must first be learned by the participants (e.g., Gómez, 20022002).

Apart from relations between specific linguistic elements, natural languages feature dependencies between syntactic classes or categories of words; for example, subject‐verb agreement in English requires subjects and verbs to agree for number: singular subjects must appear with singular verbs, regardless of the specific identity of the nouns and verbs involved. In these cases, it is necessary to both learn which category a specific word belongs to and to also learn the relationships between these categories. This can be assessed using an artificial grammar of the form “{A}X{B},” where {A} and {B} represent two sets of stimuli and therefore any stimulus from set {A} predicts any {B} category stimulus (Fig. 22iii,iv). To limit the requirement that participants must learn the categories associated with many different stimuli, some studies have used sets of stimuli in which perceptual cues denote category membership (see Fig. 22iii). For example, all {A} stimuli might be nonsense syllables containing the vowel “*i*,” while {B} stimuli contain “*o*” vowels, thus adding a clear perceptual cue to the stimulus categories. A participant would be required to first recognize that there are two (perceptually different) categories of stimuli, and then to recognize and learn the nonadjacent dependency between them (in this case, “*_i*” syllables predict “*_o*” syllables; see Fig. 22iii). This approach has been used to assess the learning of center embedded structures, which include nonadjacent dependencies (see Fig. 11v) in humans and nonhuman animals (Bahlmann, Schubotz, & Friederici, 20082008; Fitch & Hauser, 20042004; Friederici, Bahlmann, Heim, Schubotz, & Anwander, 20062006). One advantage of this approach is that once participants have learned these relationships, they may be able to generalize to novel stimuli as long as these can also be categorized using the perceptual cue.

Clear operationalized descriptions of specific types of nonadjacent dependencies (Fig. 22) may help us to understand how different types of dependencies are learned and processed across different populations and species. This clarity may be important in understanding the developmental and evolutionary origins of abilities that are critical to language.

## 3 Nonadjacent dependency learning in adults, infants, and nonhuman animals

In the previous section, we summarized several different types of nonadjacent dependencies found in natural language and how they have been studied using artificial grammar learning tasks. Next, we review how human adults and infants and nonhuman animals have been tested using these paradigms to better understand how different types of dependencies (Figs. 11 and 22) are learned and how these might vary across development and evolution.

### 3.1 Human adults and infants

Infants as young as 7 months old readily notice dependencies between identical nonadjacent items in a sequence (e.g., “*ga* *po* *ga*” vs. “*ga* *po* *bi*,” represented as “AXA” in Fig. 22i) (Gerken, 20062006; Gervain & Werker, 20132013; Marcus, Vijayan, Rao, & Vishton, 19991999). As we discussed above, these identity relations might be relatively easy to recognize because they do not require the participant to learn an arbitrary relationship between two different stimuli (as in Fig. 22ii,iii).

Many studies have also assessed the learning of dependencies between arbitrarily related stimuli, which might be relevant to a wider range of nonadjacent dependencies seen in natural languages. Arbitrary item‐based dependencies between adjacent elements appear to be easily learned from shortly after birth (e.g., Aslin, Saffran, & Newport, 19981998; Kudo, Nonaka, Mizuno, Mizuno, & Okanoya, 20112011; Perruchet & Pacton, 20062006; Saffran, Johnson, Aslin, & Newport, 19991999; Saffran et al., 19961996; Teinonen, Fellman, Näätänen, Alku, & Huotilainen, 20092009). Furthermore, several studies have found that both adults and infants as young as 3 months old show learning of nonadjacent dependencies of the form “AXB” (e.g., Citron, Oberecker, Friederici, & Mueller, 20112011; Frost & Monaghan, 20162016; Gómez, 20022002; Gómez & Maye, 20052005; Marchetto & Bonatti, 20132013; Mueller, Friederici, & Mannel, 20122012; Mueller, Oberecker, & Friederici, 20092009; Pena et al., 20022002; van den Bos et al., 20122012; Vuong et al., 20162016). However, as we discuss below, subsequent work has revealed important limits on the human capacity for learning nonadjacent dependencies and identified a number of additional cues that might emphasize these relationships and aid learning (see below and Fig. 33).

**Figure 3:**
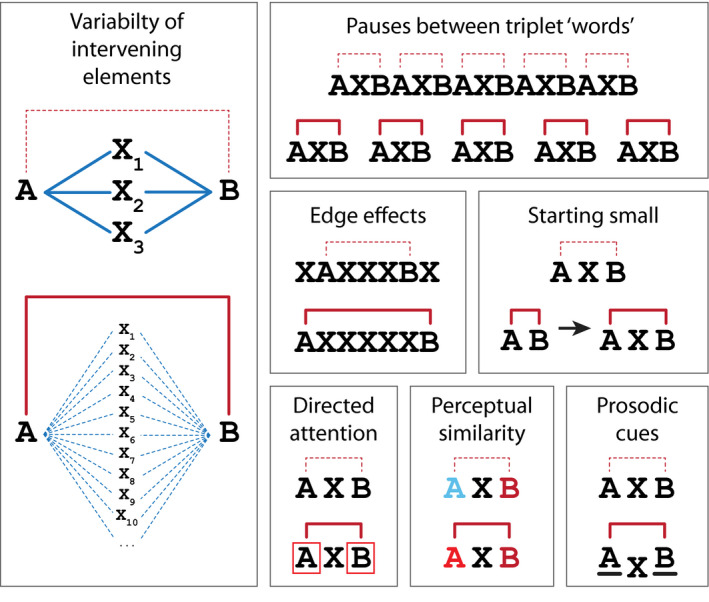
Different cues aiding nonadjacent dependency learning. The learning of nonadjacent dependencies can be improved in a number of ways. These include introducing additional variability into the possible intervening elements, thus emphasizing the nonadjacent dependency (e.g., Gómez, 20022002); adding pauses within streams of stimuli to denote “word” boundaries and the nonadjacent dependencies within them (e.g., Pena et al., 2002); positioning dependent stimuli on the periphery of sequences (e.g., Endress et al., 20092009); initially learning the dependency between adjacent items, before introducing intervening elements (e.g., Lany & Gómez, 20082008); directing attention toward dependent elements (e.g., Pacton & Perruchet, 20082008); using perceptually similar dependent elements (e.g., Newport & Aslin, 20042004); or the addition of prosodic cues that differentiate the dependent elements from the intervening stimuli (e.g., Grama et al., 20162016). All of these different cues emphasize the relationships between nonadjacent elements and facilitate learning.

#### 3.1.1 Variability and the detection of predictable dependencies

Gómez (20022002) showed that both adults and 18‐month‐old infants were able to detect a nonadjacent dependency between the first and last items of a three‐element sequence “AXB,” where the dependency involved an arbitrary association between items (e.g., initial “*pel*” predicted final “*rud*”). However, in both groups learning only occurred when a large number of different nonsense words were presented in the second position in the sequence, making the adjacent dependencies between the first and second, and second and third elements highly unpredictable (Fig. 33). When a smaller number of “X” elements was used, participants appeared to try to learn the (uninformative) adjacent relationships and failed to learn the nonadjacent dependency (e.g., Gómez, 20022002; Gómez & Maye, 20052005). This suggests that, while both adults and infants are able to implicitly learn nonadjacent dependencies, in some cases they may fail to do so if more salient adjacent cues are present. However, in some cases both adjacent and nonadjacent dependencies may be learned simultaneously (Romberg & Saffran, 2013b; Wang & Mintz, 20182018), at least in some participants (see Milne, Petkov, & Wilson, 20172017; Wilson et al., 20152015), suggesting that the processes involved in learning these two types of relationships are not inherently antagonistic.

#### 3.1.2 Perceptual similarity between dependent stimuli

A key cue which appears to facilitate the learning of nonadjacent dependencies is perceptual similarity between the “A” and “B” elements that differentiate them from the intervening “X” elements (Fig. 33; Creel, Newport, & Aslin, 20042004; Newport & Aslin, 20042004; Onnis, Monaghan, Richmond, & Chater, 20052005). For example, in Gómez (20022002), the “A” and “B” elements were represented by monosyllabic nonsense words, and the “X” items were all disyllabic, potentially aiding the learning of the nonadjacent dependency (Gómez, 20022002). Similarly, Newport and Aslin (20042004) showed that adults could learn dependencies between similar nonadjacent segments (e.g., between acoustically similar consonants separated by vowel sounds) but could not learn nonadjacent dependencies between arbitrarily related syllables (e.g., where initial “*ba*” predicted final “*te*”). When no perceptual similarities are present between the “A” and “B” stimuli, adjacent dependencies are easily learned, while there is often no evidence of nonadjacent dependency learning under these conditions (Gebhart et al., 20092009; Onnis et al., 20052005). Relatedly, the addition of prosodic cues emphasizing the relationship between the “A” and “B” elements aids the learning of nonadjacent dependencies (Grama, Kerkhoff, & Wijnen, 20162016). These studies suggest phonological or perceptual similarity acts as a cue facilitating the perceptual grouping of these nonadjacent elements and thus the learning of these dependencies (Newport & Aslin, 20042004).

#### 3.1.3 Edge effects

An additional cue that might draw attention to nonadjacent elements is positioning them at the start and the end of a sequence, as in “AXB” (Fig. 33). For example, Pena et al. (2002) presented adult participants with a continuous stream of speech stimuli grouped into trisyllabic “words” containing nonadjacent dependencies between the first and last syllables. They showed that these nonadjacent dependencies could only be learned if a brief 25 ms pause was added between each triplet “word,” thus emphasizing the word boundaries (Marchetto & Bonatti, 20132013; Pena et al., 20022002); but also see Onnis et al, 20052005). When combined with prosodic information, pauses have also been shown to aid the learning of center embedded structures, which include nonadjacent dependencies (Fig. 11vi), within a speech stream (Mueller, Bahlmann, & Friederici, 20102010). Endress and Mehler (20092009) tested the importance of these “edge effects” directly, presenting adult participants with sequences in which elements “A” and “B” occurred either at the edges of a string (e.g., “AXYZB”) or within a sequence (e.g., “XAYBZ”). They found that while participants were sensitive to the reversal of the positions of the “A” and “B” elements (e.g., “BXYZA”) in either condition, they were only sensitive to the dependency between specific “A” and “B” elements when they occurred at the edges of the sequences.

Wang, Zevin, and Mintz (20172017) showed that nonadjacent dependencies can be learned even when embedded in a continuous sequence of words, with no pauses or other signal‐driven edge cues. They first exposed participants to a continuous natural language stream in which there was a sentence boundary every four words. This entrained subjects to parse subsequent material into periodic subsequences. Seamlessly following this pre‐exposure, subjects heard a continuous stream of an artificial language containing nonadjacent “A_B” dependencies. Participants learned the nonadjacent dependency when the “A” and “B” elements appeared within a subsequence, but not if the entrained segmentation placed the dependent elements in different subsequences. Thus, top‐down cues (i.e., the entrained rhythm) can facilitate the detection of nonadjacent dependencies, in the absence of surface‐level cues such as pauses.

#### 3.1.4 Attentional effects

The presence of pauses between triplets of syllables, dependent elements appearing at peripheral positions within a string, or linguistic entrainment appear to direct attention toward the dependent elements, thus facilitating the learning of nonadjacent dependencies. Pacton and Perruchet (20082008) found that when adult participants' attention was actively directed towards either adjacent or nonadjacent elements within a numerical sequence (by asking them to perform mathematical operations on adjacent or nonadjacent pairs of numbers), only dependencies between attended stimuli were learned. When the same task was performed with no attentional requirements, participants implicitly learned relationships between adjacent items but failed to notice the nonadjacent dependencies (Pacton et al., 20152015). These studies, along with others (e.g., de Diego‐Balaguer, Martinez‐Alvarez, & Pons, 20162016; Friederici, Mueller, Sehm, & Ragert, 20132013; Toro, Sinnett, & Soto‐Faraco, 20112011), support the notion that attention facilitates the learning of nonadjacent dependencies.

#### 3.1.5 Incremental increases in complexity: Starting small

Nonadjacent dependency learning can be facilitated by initially learning the dependencies between items in adjacent sequence positions, before adding intervening elements. For example, 12‐month‐old infants were shown to be sensitive to nonadjacent dependencies, but only when they were first exposed to the same “A” and “B” items in adjacent positions in a sequence (Lany & Gómez, 20082008), before testing with nonadjacent “AXB” sequences. Similarly, hierarchical relationships, which include nonadjacent dependencies (Fig. 11vi), have been shown to be learned better by adult participants when they are initially presented as pairs of adjacent items (Lai & Poletiek, 20112011; Rey, Perruchet, & Fagot, 20122012). These studies suggest that part of the challenge of learning nonadjacent dependencies might stem not from memory demands of holding the initial element in memory, but from detecting the relationship between temporally separated stimuli in the first place.

Taken together, this research suggests that human adults and infants are able to learn dependencies between nonadjacent elements, but that this learning strongly benefits from additional cues that highlight these elements or the relationships between them. These cues emphasize the dependencies by bringing the nonadjacent elements together—physically, perceptually, or attentionally (see Fig. 33).

While nonadjacent dependency learning is required for language learning and processing, it also applies to nonlinguistic material (e.g., Creel et al., 20042004; Endress, 20102010; Endress & Wood, 20112011; Gebhart et al., 20092009; Pacton & Perruchet, 20082008), suggesting that this ability might not be domain‐specific or restricted to language. In the next section, we will discuss evidence of nonadjacent dependency learning in nonhuman animals, how these abilities relate to those observed in human adults and infants, and how this might inform us about the evolutionary origins of these abilities.

### 3.2 Nonhuman animals

A number of studies have demonstrated that many nonhuman animal species can learn adjacent relationships (for recent reviews, see Santolin & Saffran, 20172017; Wilson, Marslen‐Wilson, & Petkov, 20172017; ten Cate, 20182018). However, there is also evidence that some nonhuman animals are able to learn nonadjacent dependencies in certain situations.

Nonadjacent dependency learning based on identity relations across a range of stimuli (i.e., “AXA” in Fig. 22i) has been demonstrated in squirrel monkeys (Ravignani et al., 20132013) and chimpanzees (Ravignani & Sonnweber, 20172017; Sonnweber et al., 20152015). These studies suggest that some animals are able to detect nonadjacent dependencies, at least between identical items. Several studies have used sequences of the form “ABA,” in which the first and last element of a sequence are identical (Murphy, Mondragon, & Murphy, 20082008; Spierings & ten Cate, 20162016). However, these experiments typically assess the ability to differentiate between “ABA” sequences and those of a different form (e.g., “ABB” or “AAB”), which can be identified on the basis of adjacent repetitions and do not provide evidence for learning a nonadjacent dependency.

A number of studies have tested whether nonhuman animals are able to learn nonadjacent dependencies between two different stimuli, using grammars of the form “AXB” (Fig. 22ii). However, as in humans, these studies have produced somewhat mixed results. Newport et al. (20042004) conducted a study in tamarin monkeys based on a similar human experiment (Newport & Aslin, 20042004). The human participants failed to learn nonadjacent dependencies between different syllables but were able to detect dependencies over both vowels and consonants (see above, Newport & Aslin, 20042004). By contrast, tamarins were able to learn the dependencies based on syllables and vowels, but not consonants, suggesting that the vowel sounds (including within syllables) might be particularly salient to the monkeys (Newport et al., 20042004). Recently, nonadjacent dependency learning has been demonstrated in the visual modality in tamarins (Versace et al., 20172017) and baboons (Malassis, Rey, & Fagot, 20182018), suggesting that these abilities are not limited to the auditory domain. de la Mora and Toro (20132013) presented rats with sequences of nonsense words of alternating consonants and vowels of the form “CVCVCV,” containing nonadjacent dependencies between either the vowels or the consonants. Rats detected the dependencies in both cases (de la Mora & Toro, 20132013, although see Toro & Trobalón, 20052005). These studies demonstrate that at least some nonhuman animals appear to be sensitive to these types of nonadjacent dependencies, but also point to potential cross‐species differences, including between humans and nonhuman animals, in how they might be learned.

Two recent studies using mixed complexity artificial grammars, which contain both adjacent and nonadjacent dependencies, showed that macaque monkeys were able to learn relationships between adjacent stimuli in the auditory or visual modality, but they found no evidence that they were sensitive to the nonadjacent dependencies (Milne et al., 20172017; Wilson et al., 20152015). Although humans have been reported to simultaneously learn both adjacent and nonadjacent dependencies within a mixed‐complexity grammar (Romberg & Saffran, 2013a; Wang & Mintz, 20182018; Wilson et al., 20152015) (although not in Milne et al., 20172017), it is likely that in these studies the presence of salient adjacent relationships prevented the monkeys from learning these nonadjacent dependencies (as in infants in Gómez, 20022002). However, a recent EEG experiment (Milne et al., 20162016) reported that violations of nonadjacent dependencies evoked similar brain potentials in macaques as had previously been reported in humans using identical stimuli (Mueller et al., 20122012). These results suggest that like humans, monkeys might be sensitive to nonadjacent dependencies in some conditions.

Chimpanzees' abilities to learn nonadjacent dependencies between visual stimuli have been assessed using operant training tasks. Some chimpanzees were able to learn nonadjacent dependencies between stimuli at the start and end of a sequence over variable distances (Fig. 11ii) and based on identity relations (Fig. 22i) or arbitrary associations (Fig. 22ii, Sonnweber et al., 20152015). Moreover, the presentation of structurally incongruous auditory stimuli appears to interfere with learning in the visual modality (Ravignani & Sonnweber, 20172017). These studies suggest that chimpanzees are able to learn nonadjacent dependencies between stimuli at least at the edge of visual sequences. Endress et al. (20102010) specifically assessed the salience of these “edge effects,” asking whether chimpanzees and humans could learn nonadjacent dependencies between auditory stimuli within a sequence of distracting elements. They showed that while both species were sensitive to positional effects (an “A” or “B” element occurring in an unexpected sequence position), neither humans nor chimps learned the dependency between “A” and “B” elements, embedded within a sequence (Endress et al., 20102010). Finally, Chen and ten Cate (20172017) used a “starting small” approach (Fig. 33) in which zebra finches first learned the dependency between two adjacent sequence elements (as in Lany & Gómez, 20082008). They demonstrated that, after training with stimuli of incrementally increasing complexity, the birds were capable of detecting nonadjacent dependencies both over varying distances and at different positions within the sequences.

Taken together, these studies demonstrate that the ability to learn different types of nonadjacent dependencies (Figs. 11 and 22) is not unique to humans but may be shared by some nonhuman animals. In some cases (e.g., nonhuman primates) this might suggest a common evolutionary origin of these abilities, while in other cases (e.g., songbirds) convergent evolution might have led to the independent emergence of impressive learning abilities. Evidence of similarities and differences in sequence learning across different species allows phylogenetic analysis of the evolution of these abilities, and it may further our understanding of the evolutionary origins of cognitive processes that might represent prerequisites for language processing. Moreover, if similar abilities are indeed present across species, this will allow these processes to be studied at a neurobiological level, providing insights into the mechanisms and neural computations that underpin the learning of these types of dependencies, as has been done with adjacent dependencies (Kikuchi et al., 20172017; Lu & Vicario, 20142014).

## 4 Conclusions

The learning of dependencies between adjacent stimuli is a fundamental cognitive ability, widely conserved in the animal kingdom. However, learning and processing nonadjacent dependencies, which is required in language, appears to be more difficult. Learning is aided by the addition of cues which highlight the relationship between the nonadjacent elements, bringing them closer together either physically (e.g., Lany & Gómez, 20082008), attentionally (e.g., Pacton & Perruchet, 20082008), or perceptually, via acoustic similarity (Newport & Aslin, 20042004), prosodic cues (Grama et al., 20162016), pauses between words (Pena et al., 20022002), edge effects (Endress et al., 20092009; Wang et al., 20172017) or by making the intervening adjacent relationships less salient (Gómez, 20022002). When sufficient cues are provided, nonadjacent dependency learning has been demonstrated in human adults and infants, and some nonhuman animals, suggesting that in both evolutionary and ontogenetic terms this ability appears to arise before the origins of language. However, some differences in learning are evident between human adults, and nonhuman animals (e.g., de la Mora & Toro, 20132013; Newport & Aslin, 20042004; Newport et al., 20042004; Wilson et al., 20152015), and further research will be required to determine how similarly these groups learn and process different forms of nonadjacent dependencies. Better understanding similarities and differences across these groups will benefit from carefully considering types of stimuli and dependencies involved (Figs. 11 and 22), and the perceptual cues available (Fig. 33). In this way, artificial grammar learning paradigms and sequence processing tasks offer great potential to explore both how language is learned over development and, via comparative studies, how it may have evolved.

## Funding

This work was supported by a Wellcome Trust Sir Henry Wellcome Fellowship to BW (WT110198/Z/15/Z); FWO Pegasus^2^ Marie‐Curie fellowship (12N5517N) to A. Ravignani; the European Research Council (ERC) under the European Union's Horizon 2020 research and innovation program (grant agreement 681942) to KS; DFG Forschergruppe 2253, TP1 to AK; the Netherlands Organisation for Scientific Research NWO (360‐70‐270) to FW; and the Chunked ANR‐project (#ANR‐17‐CE28‐0013‐02) to A. Rey.
